# *Proprotein Convertase Subtilisin*/*Kexin Type 9* (*PCSK9*) Inhibitor Therapy Reduces the Level of DNA Damage in Patients with Heterozygous Familial Hypercholesterolemia

**DOI:** 10.3390/ijms262110529

**Published:** 2025-10-29

**Authors:** Ewelina Woźniak, Agnieszka Woźniak, Agnieszka Pawlos, Marlena Broncel, Joanna Satała, Bożena Bukowska, Paulina Gorzelak-Pabiś

**Affiliations:** 1 Laboratory of Tissue Immunopharmacology, Department of Internal Diseases and Clinical Pharmacology, Medical University of Lodz, Kniaziewicza 1/5, 91-347 Lodz, Poland; agnieszka.wozniak@umed.lodz.pl (A.W.); agnieszka.pawlos@umed.lodz.pl (A.P.); marlena.broncel@umed.lodz.pl (M.B.); joanna.satala@umed.lodz.pl (J.S.); paulina.gorzelak@umed.lodz.pl (P.G.-P.); 2 Department of Biophysics of Environmental Pollution, Faculty of Biology and Environmental Protection, University of Lodz, Pomorska 141/143, 90-236 Lodz, Poland; bozena.bukowska@biol.uni.lodz.pl

**Keywords:** DNA damage, heterozygous familial hypercholesterolemia, PCSK9 inhibitors

## Abstract

Heterozygous familial hypercholesterolemia (HeFH) is a common autosomal dominant genetic disease (1:250) characterized by elevated LDL-C. Patients with HeFH are at increased risk of premature atherosclerosis and have at least a 10-fold greater chance of cardiovascular disease (CVD). The present study examines the effect of PCSK9 inhibitor treatment (iPCSK9: arilocumab or evolocumab) on DNA damage in HeFH patients. Fifty-six patients were studied, with a normolipidemic group (control; *n* = 20) and patients with HeFH (study group; *n* = 36). DNA damage was determined by alkaline comet assay and PCSK9 protein level by ELISA. PCSK9i treatment was found to be associated with lower DNA damage, Lp(a), PCSK9, and lipid profile compared to before treatment. However, 16 of 36 patients still had Lp(a) values above 125 nmol/L, and reduced Lp(a) did not correlate with reduced DNA damage. Reduced PCSK9 demonstrated a moderately positive correlation (r = 0.48) with reduced DNA damage. PCSK9i therapy reduces the level of DNA damage in HeFH patients, regardless of the type of inhibitor. While our findings confirm that PCSK9 treatment can reduce DNA damage, the mechanism remains unclear.

## 1. Introduction

Heterozygous familial hypercholesterolemia (HeFH) is the most common genetic disorder in humans, caused mainly by mutations in the LDLR, APOB, or PCSK9 genes. Its prevalence is estimated to range from 1:200 to 1:300 in the global population and is approximately 1:250 in Poland, i.e., approximately 150,000 people [1,2]. Unfortunately, only about 5% of patients who suffer from HeFH have been properly diagnosed, and an even smaller number receive optimal treatment [3].

The condition is characterized by very high plasma levels of low-density lipoprotein cholesterol (LDL-c), ranging from 190 to 400 mg/dL. Patients with heterozygous FH (HeFH) may develop cardiovascular disease (CVD) [1] and are at least 10 times more likely to develop ASCVD compared to the general population. In turn, coronary artery disease (CAD) is the most common cause of death in this group (frequency is 60%) [4].

Patients with HeFH require quick diagnosis and the initiation of effective lipid-lowering treatment, which may require long-term administration. However, the long-term impact of long-term lipid-lowering treatment remains unclear.

In HeFH patients who do not respond effectively to maximal tolerated statin plus ezetimibe, it is recommended to change to a PCSK9 inhibitor (PCSK9i) [3]. The major function of PCSK9i is to prevent the interaction between PCSK9 and LDL-C receptors, resulting in the increased uptake of cholesterol-rich atherogenic lipoproteins, i.e., low density lipoproteins (LDL), from the bloodstream [5]. Two large clinical trials (FOURIER and ODYSSEY OUTCOMES) have shown that evolocumab and alirocumab have a beneficial effect on cardiovascular outcomes when added to statin therapy. Both evolocumab and alirocumab are believed to be completely safe and demonstrate no difference in safety compared to the placebo [6,7].

It is possible that PCSK9 inhibitors may have a beneficial pleiotropic effect on patients with HeFH, in a similar way to statins. In recent years, PCSK9I drugs have demonstrated a number of pleiotropic properties in addition to their lipid-lowering ability. PCSK9i treatment reduces the production of pro-inflammatory cytokines, significantly lowers LDL levels, and reduces LDL receptor-mediated platelet activation; it also possesses anti-autophagic, antioxidant [8], anti-atherosclerotic, and antineoplastic effects, and it has been found to stabilize atherosclerotic plaques and influence the course of bacterial infection [9,10,11].

Cardiovascular disease is characterized by endothelial cell damage, which is mainly caused by lipid peroxidation exacerbated by the action of ROS. The end-products of lipid peroxidation induce the formation of DNA bulky adducts, leading to genome instability. Woźniak et al. (2024) [12] reported higher levels of DNA damage in patients with HeFH compared to normolipidemic patients, with greater damage noted in those with ASCVD. Oxidative stress markers were also found to be increased in patients; however, only oxLDL was elevated in those with both ASCVD and HeFH, and its level correlated with DNA damage. Furthermore, even following maximally tolerated lipid-lowering statin plus ezetimibe treatment, the HeFH patients did not achieve their therapeutic goals. Interestingly, no differences in damage were found between the different types of hypolipemic therapy used [12].

Assuming that iPCSK9 has pleiotropic effects, the aim of the present study was to determine the possible impact of treatment on reducing the level of DNA damage in HeFH patients.

## 2. Results

The study group (*n* = 36 patients) consisted of HeFH patients treated with the PCSK9 inhibitors evolocumab and alirocumab; of these participants, 14 (38.9%) were men, and 22 (61.1%) were women. The median age was 57 years (IQR, 45–68 years). The control group consisted of 20 patients with normolipidemia. This group was significantly younger than the study group (*p* = 0.0177); however, no significant correlations were found between age and the studied parameters. No significant differences in sex ratio were observed between groups (*p* > 0.05).

In the study group, the following parameters were analyzed at baseline, i.e., before treatment with iPCSK9, and again after six months of treatment: lipid profile, Lp(a), creatinine, eGFR, creatine kinase (CK), ALT, hsCRP, FT4, TSH, DNA damage, and PCSK9 level. The creatinine, eGFR, creatine kinase (CK), ALT, hsCRP, FT4, and TSH levels remained normal throughout the study period and did not change from baseline. The clinical data, including sex, age, pre-existing medical conditions, FH diagnosis, characteristics features, and lipid-lowering treatment, are given in Table 1.

### 2.1. HeFH Patients Demonstrated Lower Levels of DNA Damage After PCSK9i Treatment Compared to Before Treatment

**Figure 1:**
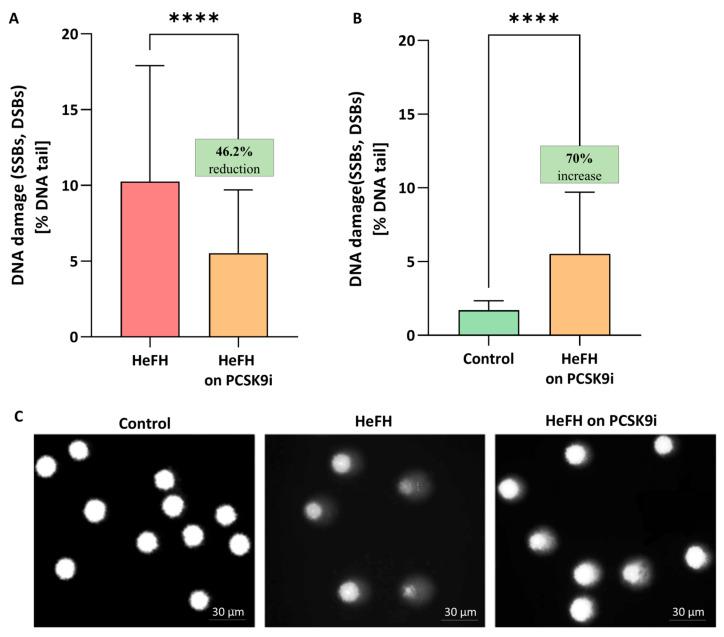
(**A**) Percentage of DNA in the comet tail from the DNA damage (SSBs, DSBs, and ALSs) of patients with HeFH before and after treatment with PCSK9i (*n* = 36). For controls, % DNA damage in the tail is 1.71 [1.35–2.34]. (**B**) Percentage of DNA in the comet tail from the DNA of normolipidemia (*n* = 20) and patients with HeFH after treatment with PCSK9i. In the box shown, % reduction/increase is shown as a median. One hundred images (comets) were randomly selected from each sample, and the median value of DNA in the comet tail was taken as an index of DNA damage (expressed in percent). Each experiment included a positive control (PC) treated with hydrogen peroxide at 20 μM (% DNA tail: 43.1 ± 7.5). (**C**) Selected photographs of DNA (comets) of patients. The photos were obtained using a fluorescent microscope with 200× magnification. Significant differences are indicated by **** *p* < 0.0001. Statistical analysis was conducted using the Wilcoxon test (**A**) and the Mann–Whitney test (**B**).

Significantly greater DNA damage, reflected as SSBs, DBSs, and alkali labile sites (ALSs), was noted in the HeFH patients (10.04 [5.79–17.2] % DNA damage) compared to normolipidemic controls (1.71 [1.35–2.34] % DNA damage). PCSK9i treatment resulted in lower levels of DNA damage (SSBs, DBSs and ALSs) (5.52 [1.84–9.71] % DNA damage in the comet tail; this value differed significantly between groups (*p* < 0.0001). The percentages of DNA in the comet tail of the HeFH patients, before and after PCSK9i treatment, are shown in Figure 1A, and selected comets are shown in Figure 1C. Although PCSK9i therapy significantly reduced DNA damage, the level remained higher than control values (1.71 [1.35–2.34] vs. 5.52 [1.84–9.71] % DNA damage (*p* < 0.0001)) (Figure 1B).

### 2.2. Both Evolocumab and Alirocumab Significantly Reduce DNA Damage Levels

**Figure 2:**
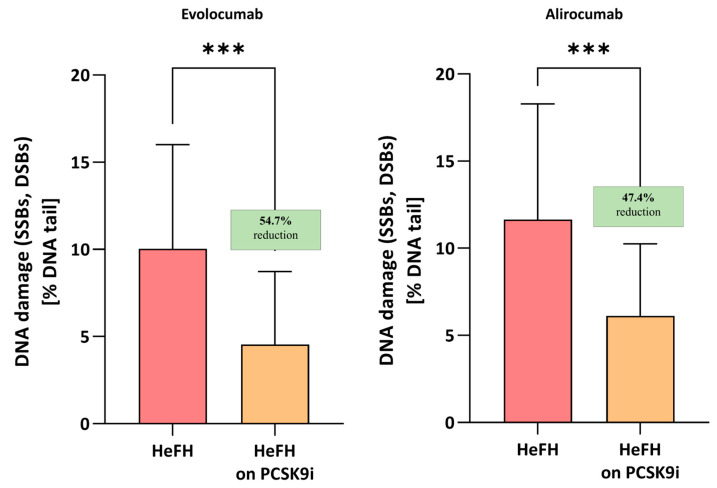
Percentage of DNA in the comet tail from the DNA damage (SSBs, DSBs, and ALSs) of patients with HeFH before and after treatment with evolocumab (*n* = 19) and alirocumab (*n* = 17). In the boxes shown, % reduction is shown as a median. One hundred images (comets) were randomly selected from each sample, and the median value of DNA in the comet tail was taken as an index of DNA damage (expressed in percent). Significant differences are indicated by *** *p* < 0.001. Statistical analysis was conducted using the Wilcoxon test.

Evolocumab treatment was associated with a significant reduction in DNA damage (SSBs, DBSs, and ALSs), i.e., 4.55 [2.82–8.72] % DNA damage, compared to before treatment, i.e., 10.04 [7.61–16.01] % DNA damage; this difference was statistically significant (*p* < 0.001). Alirocumab also significantly reduced the level of DNA damage in patients (5.96 [3.47–10.26] vs. 11.64 [5.9–18.28] % DNA damage) with a significance level of *p* < 0.005. No significant differences were found between the two inhibitors (*p* = 0.7072). The percentages of DNA in the comet tail and the reduction in DNA damage among HeFH patients during treatment are shown in Figure 2.

### 2.3. Significantly Lower LDL, Non-HDL, Total Cholesterol, TG, and Lp(a) Levels Are Significantly Lower After Treatment with PCSK9 Inhibitors

**Figure 3:**
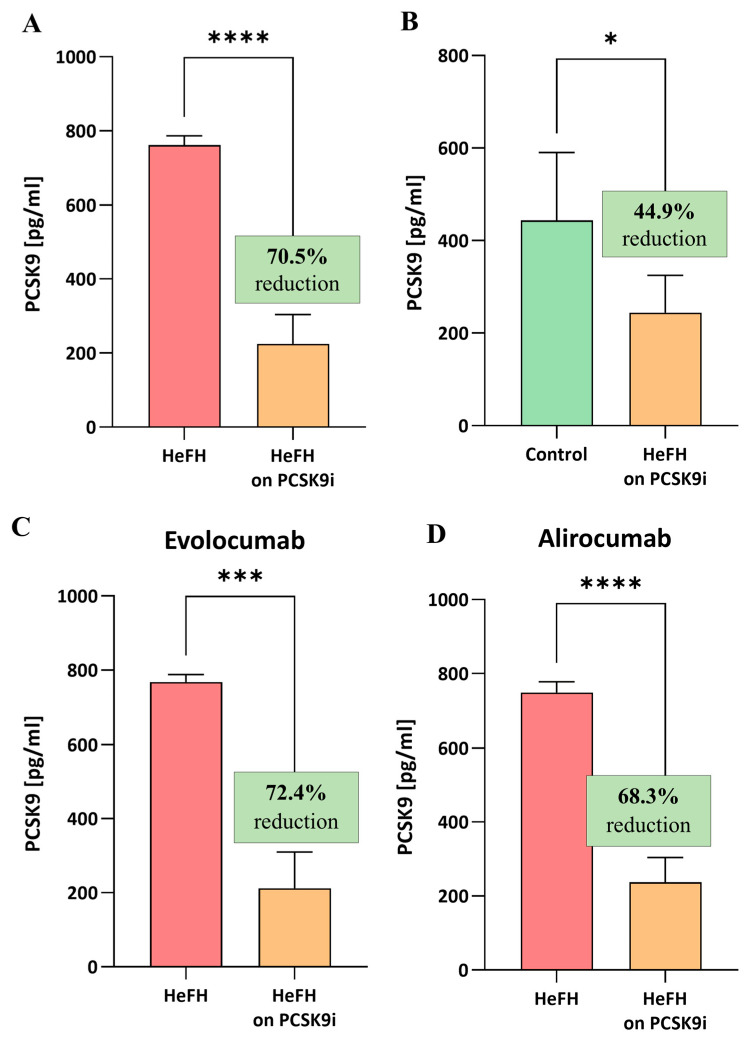
(**A**) Serum levels of PCSK9 protein in patients with HeFH before and after treatment with PCSK9 inhibitors (*n* = 36). (**B**) Serum levels of PCSK9 protein in controls (*n* = 20) and patients with HeFH after treatment with PCSK9 inhibitors. (**C**) PCSK9 level of individual patients before and after treatment with evolocumab (*n* = 19). (**D**) PCSK9 level of individual patients before and after treatment with alirocumab (*n* = 17). In the box shown, % reduction is shown as a median. Significant differences are indicated by **** *p* < 0.0001; *** *p* < 0.001; and * *p* < 0.05. Statistical analysis was conducted using the Wilcoxon test (**A**,**C**,**D**) and the Mann–Whitney test (**B**).

**Figure 4:**
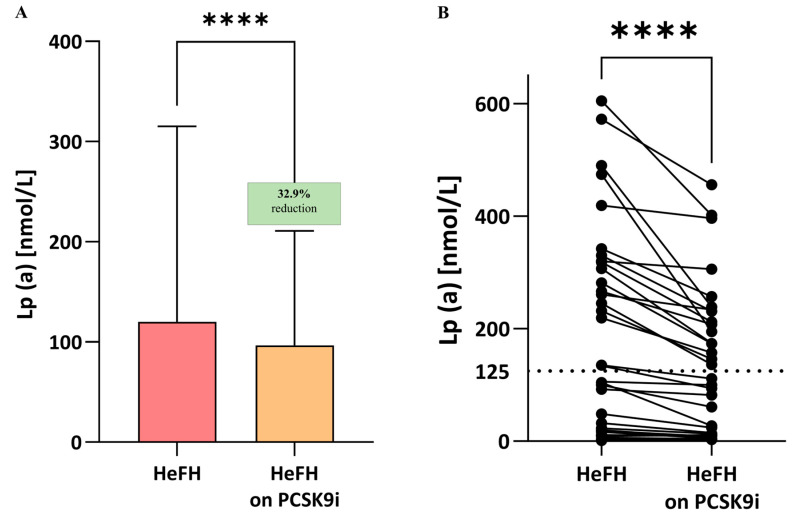
(**A**) Serum levels of Lp(a) in patients with HeFH before and after treatment with PCSK9 inhibitors (*n* = 36). (**B**) Lp(a) level of individual patients before and after treatment with PCSK9i. The serum level of Lp(a) in controls is 10.7 nmol/L. In the box shown, % reduction is shown as a median. The dotted line indicates the target value of Lp(a). Significant differences are indicated by **** *p* < 0.0001. Statistical analysis was conducted using the paired student *t*-test.

Significantly lower LDL-C, non-HDL, total cholesterol, TG, and Lp(a) levels were observed compared to the start of treatment; the significance was *p*  < 0.0001 for LDL, non-HDL, total cholesterol, and Lp(a) and *p* < 0.005 for triglycerides (Table 2, Lp(a) also Figure 3A,B). In 16 patients, Lp(a) values remained above 125 nmol/L after PCSK9i treatment (Figure 4A,B).

### 2.4. Serum Levels of PCSK9 Protein Are Significantly Lower in Patients After Treatment with PCSK9 Inhibitors

The HeFH patients demonstrated significantly lower levels of PCSK9 protein after treatment (evolocumab or alirocumab), i.e., 224.7 [112.9–303.8] pg/mL, compared to the start of treatment, i.e., 761.6 [427–786.6] pg/mL, (*p* < 0.0001). The reduction in PCSK9 protein level did not differ significantly between evolocumab and alirocumab (*p* = 0.7319). The serum levels of PCSK9 protein in the HeFH group before and after treatment are shown in Figure 3A,C.

At the end of treatment, the levels of PCSK9 protein in the HeFH group were significantly lower than those in the normolipidemic group, as follows: 244.4 [121.9–324.9] pg/mL vs. 443.8 [183.7–590.3] pg/mL (*p* = 0.0301; Figure 3B).

### 2.5. DNA Damage Positively Correlates with PCSK9 Protein Levels After PCSK9i Therapy

**Figure 5:**
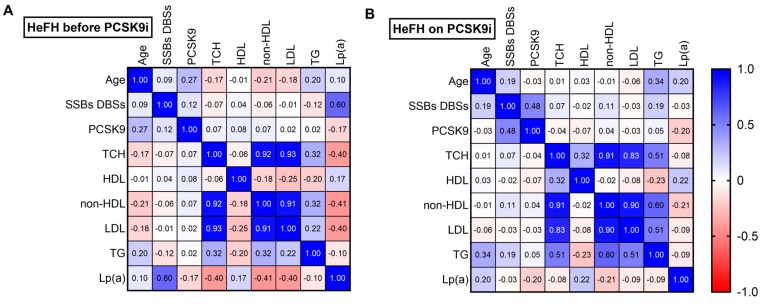
Spearman’s correlation heatmap for (**A**) patients with HeFH before treatment with PCSK9 inhibitors and (**B**) patients with HeFH after treatment with PCSK9 inhibitors (*n* = 36). Blue squares indicate significant positive correlations (r > 0.5, *p* < 0.05), white squares indicate non-significant correlations (*p* > 0.05), and red squares indicate significant negative correlations (r < −0.5, *p* < 0.05).

Among the HeFH patients, a fairly strong positive correlation was noted between DNA damage and Lp(a) level before treatment (r  =  0.60; *p*  <  0.05) (Figure 5A). After treatment, a moderately positive correlation was found between DNA damage and PCSK9 protein (r  =  0.48; *p*  <  0.05) (Figure 5B).

## 3. Discussion

It has been proposed that PCSK9 inhibitors may exert antioxidant effects on endothelium exposed to IL-6 during CVD. This may account for the reduced levels of DNA damage observed in patients after PCSK9i treatment [8].

Our present findings indicate that DNA damage is reduced in HeFH patients receiving PCSK9 inhibitors; in addition, no significant differences in DNA damage reduction were observed between the two PCSK9is. It has previously been found that patients with HeFH demonstrate higher levels of DNA damage than those with normolipidemia. Despite receiving maximally tolerated lipid-lowering therapy (statin plus ezetimibe), some HeFH patients do not achieve their therapeutic goals, and the type of hypolipemic therapy does not appear to have any effect on the level of DNA damage [12].

Preventing DNA damage may be associated with potential long-term benefits associated with reductions in the risk of cardiovascular disease and cancer. One source of DNA damage, manifested as single-strand breaks (SSB) and double-strand breaks (DSB), can be the ROS generated in familial hypercholesterolemia; indeed, ROS can exacerbate lipid peroxidation, resulting in endothelial cell damage, a characteristic feature of cardiovascular disease. The end-products of lipid peroxidation induce the formation of DNA bulky adducts, leading to genome instability and the development of neoplasia. While oxidative DNA damage is typically countered by increased activity of the base excision repair (BER) pathway, oxLDL lowers the level of its constituent enzymes, including those responsible for the removal of 8-oxoguanine (8-OH-Gua) [13,14]; as such, oxidative stress commonly results in the accumulation of 8-OH-Gua. In advanced atherosclerotic changes, high levels are observed in vascular smooth muscle cells, macrophages, and endothelial cells [15,16]. Our previous work confirms that patients with HeFH demonstrate greater DNA damage, increased oxLDL and anti-oxLDL levels, and lower total plasma antioxidant capacity than those with normolipidemia [12]. It is likely that PCSK9i treatment reduces DNA damage in patients with HeFH by lowering ROS levels.

The main goal of using PCSK9 inhibitors is to reduce serum LDL-C level. Our findings confirm those of previous studies indicating that PCSK9i treatment reduces circulating PCSK9 level; this change also moderately (r = 0.48) correlates with a reduction in the level of DNA damage [17,18]. However, there is currently little data regarding the impact of PCSK9 inhibitors on the reduction of DNA damage. It is possible that PCSK9i may indirectly attenuate DNA damage by reducing oxidative stress. An increasing body of evidence supports the role of PCSK9i in mitigating inflammation and oxidative stress through pathways other than those associated with LDL receptor modulation, although the exact mechanisms remain unclear. Numerous studies have implicated PCSK9 in the induction of oxidative stress, the promotion of chronic inflammation, and the interplay between these processes, particularly in the pathogenesis of atherosclerosis [19,20]. Ding et al. provided compelling evidence for a bidirectional crosstalk between PCSK9 expression and ROS generation, noting that, under conditions of low shear stress, both vascular smooth muscle cells and endothelial cells exhibit increased PCSK9 expression and ROS production [21]. PCSK9 can stimulate oxLDL formation via NOX-2 [22]. Additionally, high PCSK9 levels were found to be associated with a ROS-mediated pathway in patients with atrial fibrillation [23]. PCSK9 inhibitors have been found to possess intrinsic anti-inflammatory, anti-autophagic, and antioxidant properties in human aortic endothelial cells (TeloHAEC); while they are believed to act by influencing the SIRT3 protein, a mitochondrial deacetylase that can increase the activity of antioxidant enzymes, their effect on pro-antioxidant parameters requires further research [8]. PCSK9 inhibitors have been found to directly affect the level of oxidative stress markers, such as ferric reducing antioxidant power (FRAP), lower hydroperoxide and malondialdehyde (MDA) levels, and increased cell viability in response to H_2_O_2_ [24]. Furthermore, in vivo models have shown that evolocumab treatment reduced the level of reactive oxygen species in the aorta and reduced the level of the oxidative damage marker 8-hydroxy-2′-deoxyguanosine (8-OHdG) [25]. Recent evidence [26,27,28] indicates that PCSK9 inhibitors may also exert prophylactic cardioprotective effects in patients receiving ANT-based chemotherapies. As the mechanisms of PCSK9 antagonism likely overlap with pathways involved in ANT-induced cardiotoxicity, these agents may hold potential for preventing chemotherapy-related cardiac damage.

As noted previously, PCSK9i treatment significantly reduced the concentrations of LDL-C, Non-HDL, TC, and TG, and this was accompanied by a significant increase in HDL-C concentration. At the end of the study, all patients achieved the LDL-C treatment target. After therapy with PCSK9 inhibitors, LDL-C was reduced by 66% compared to before treatment. Similarly, randomized controlled clinical trials (FOURIER and ODYSSEY OUTCOMES) found that both evolocumab and alirocumab treatments reduce LDL-C levels by 50% or greater, and both have a beneficial effect on cardiovascular outcomes when added to statin therapy [6,7].

Unlike statins, PCSK9 inhibitors are able to reduce Lp(a) levels [7]. Indeed, the Lp(a) values were significantly lower in the present study and anticipated reduction with PCSK9 inhibitors [6,7]. It is worth noting that, although PCSK9i treatment resulted in lower Lp(a) levels in all patients, 16 out of 36 maintained Lp(a) values above the target value, i.e., 125 nmol/L, and no relationships were found between Lp(a) and DNA damage. Lp(a) levels of 75–125 nmol/L (30–50 mg/dL) are associated with moderate cardiovascular risk, according to the 2021 Polish Lipid Society Guidelines [24].

Our previous work demonstrated a fairly strong positive correlation between increased DNA damage and elevated Lp(a) levels in HeFH patients prior to lipid-lowering therapy. This relationship was confirmed in the present study (r = 0.60; *p* < 0.05). However, no correlation was found between Lp(a) and DNA damage in HeFH patients following PCSK9 inhibitor treatment, during which both Lp(a) levels and DNA damage were reduced. This may be related to the failure to achieve target Lp(a) levels, which could be due to genetic variation or insufficient follow-up duration; however, this remains to be verified in subsequent studies on larger groups of patients.

The literature data confirm that PCSK9i treatment reduces Lp(a) levels; however, the maximum reduction is approximately 30%, which, in most cases, is insufficient to achieve Lp(a) values below 125 nmol/L [29]. Patients in all quartiles of baseline Lp(a) level experienced similar reductions in relative risk for MACE with alirocumab compared to the placebo, but patients from the higher baseline Lp(a) quartiles demonstrated greater absolute risk reduction compared to those from the lower quartiles. Overall, after adjusting for the effect of LDL reduction, Lp(a) reduction significantly contributed to lower MACE risk, but this contribution was minimal in the lower quartiles of baseline Lp(a) [30].

Lp(a) concentrations can be reduced by treatment with olpasiran, a small interfering RNA molecule, or pelacarsen, an antisense oligonucleotide (ASO) that inhibits apo(a) mRNA translation in hepatocytes. Administration has been found to lower Lp(a) level by approximately 80% in patients with established cardiovascular disease [31,32], which is a significant reduction in cardiovascular risk in patients with known CVD and elevated Lp(a) levels. Both olpasiran and pelacarsen are undergoing clinical trials (OCEAN[a]-DOSE-NCT04270760; HORIZON-NCT04023552).

The lack of reduction in Lp(a) levels below 125 nmol/L may also explain the lack of correlation between Lp(a) levels and DNA damage after PCSK9i treatment; indeed, a positive correlation has previously been reported between Lp(a) levels and DNA damage. Our present analysis also indicates no correlations between DNA damage and individual lipid profile parameters.

The observed reduction in DNA damage observed herein is most likely caused by the action of PCSK9i as a whole, and not only by its potential to lower the level of LDL or Lp(a) alone. Nevertheless, our findings indicate a positive correlation between DNA damage and PCSK9 protein level in HeFH patients after PCSK9i therapy.

This study has certain limitations. The first is the small size of the control and study groups, which can be attributed to the low percentage of correctly diagnosed patients in Poland (5%). However, this work is intended as a pilot for future studies with larger and more diverse cohorts, including a wider range of PCSK9i lipid-lowering treatments. Another limitation of the present study is the significant age difference between the control and study groups. Although no correlations were observed between age and the investigated parameters, it should be emphasized that age is a well-established factor that affects both DNA damage and cardiovascular risk. Finally, the mechanism explaining the reduction of DNA damage in HeFH patients following PCSK9 inhibitor treatment remains poorly defined. Although our findings suggest a potential link, further research is warranted to elucidate the underlying biological pathways. In particular, future studies should focus on the evaluation of oxidative DNA lesions, including purine- and pyrimidine-derived damage, and on the effects of PCSK9 inhibitors on oxidative stress. Further studies are needed to verify the lack of correlation between Lp(a) reduction and DNA damage in HeFH patients treated with PCSK9i, particularly in larger cohorts with baseline Lp(a) > 125 nmol/L whose levels are reduced below this threshold with therapy.

## 4. Materials and Methods

### 4.1. Patients with HeFH and Controls with Normolipidemia

The study group consisted of 36 patients who had been referred to the Lipid Clinic in Bieganski Memorial Hospital, Lodz, for hypercholesterolemia by their primary care physicians. All patients were referred from 1 April 2023 to 31 March 2024.

All patients had been diagnosed with primary familial hypercholesterolemia (HeFH) based on a score above eight points on the Dutch Lipid Clinic Network (DLCN) scale, family history, clinical interview, physical examination, and LDL-C level [30,33]. Identifying FH patients with the use of DLCNS is also recommended by ESC CVD prevention Guidelines. Therefore, in routine clinical practice, the clinical diagnosis using DLCNS is considered sufficient for both diagnostic and therapeutic decisions, including eligibility for anti-PCSK9 treatment reimbursement [30].

Among the 36 patients, 14 were diagnosed with a genetic form of HeFH based on the next-generation sequencing (NGS) of FH-related genes, i.e., mutations in the LDL-C receptor (LDLR), apolipoprotein B (APOB), or the proprotein subtilisin/kexin type 9 convertase gene (PCSK9) with confirmation by Sanger sequencing.

The exclusion criteria comprised secondary causes of hypercholesterolemia, including hypothyroidism, kidney diseases, poorly controlled diabetes, cholestasis, or the use of drugs impairing lipid metabolism.

The control group consisted of 20 patients aged 18–65 years with low cardiovascular risk (SCORE2 < 2.5% for patients aged under 50 years, or <5% for patients aged 50–69 years), LDL-C concentration < 3 mmol/L (<115 mg/dL), free of medications, and no previous chronic or acute diseases in the past three months. In addition, no abnormalities were revealed under physical examination [30].

The investigation was approved by the Bioethics Committee of the Medical University of Lodz (RNN/191/21/ KE), dated 10 January 2023. Informed consent was obtained from all participants. All methods were carried out in accordance with relevant guidelines and regulations.

### 4.2. Sample Collection and Diagnostic Laboratory Methods

The following data were collected from all participants during interviews, i.e., cases and controls: personal history of hypertension, diabetes, smoking, cardiovascular disease, chronic kidney disease, non-alcoholic fatty liver disease, pharmacological treatment, and family history of hypercholesterolemia and cardiovascular disease. All participants underwent a physical examination to identify the presence of corneal arcus and tendon xanthomas.

Peripheral whole blood samples were taken from all participants after 10 h of fasting. Peripheral blood mononuclear cells (PBMCs) and serum were then isolated by a centrifugation series using a gradient medium for lymphocyte isolation. The isolated lymphocytes were suspended in a mix (45% RPMI medium, 45% bovine serum, and 10% DMSO), then frozen at −80 °C in a box with isopropanol, and then stored in liquid nitrogen for up to 28 days.

The lipid profile, ALT, creatinine, eGFR, creatine kinase (CK), and Lp(a) levels were determined in both cases and controls. The lipid profile, consisting of total cholesterol (TCh), LDL-C, triglycerides (TG), HDL-C, and non-HDL level, was then determined by colorimetric assay. All of the biochemical assays were performed using a Cobas 6000 or 8000 system (Roche, Basel, Switzerland). All serum or plasma samples intended for the sandwich ELISA analysis were aliquoted and stored at −80 °C for later use. All biochemical measurements were performed in a central laboratory at Bieganski Hospital.

### 4.3. Enzyme-Linked Immunosorbent Assay (ELISA)

The serum concentrations of PCSK9 protein were determined in the control and research groups by a sandwich ELISA kit according to the manufacturer’s instructions (Catalog# SEE189Hu; Cloud-Clone Corp. (Katy, TX, USA). Serum PCSK9 levels were presented as ng/mL. The minimum detectable dose of PCSK9 is typically less than 1.21 ng/mL. Absorbance was measured at a wavelength of 450 nm using the ELISA ST-360 microplate reader (by Shanghai Kehua Bio-Engineering Co., Shanghai, China). The intra-assay coefficient was 10%, and the inter-assay coefficient of variations was <12%. In this method, values were calculated quantitatively by drawing graphs with standards.

### 4.4. Particle-Enhanced Immunoturbidimetric Assay

The levels of lipoprotein(a) in human serum were quantitatively determined with a Cobas c system (Roche/Hitachi, Indianapolis, IN, USA), using Tina-quant Lipoprotein (a) Gen.2.; LPAM2: ACN 8724 (Roche Diagnostics, Indianapolis, IN, USA). Human lipoprotein (a) was allowed to agglutinate with latex particles coated with anti-Lp(a) antibodies, and the precipitate was determined turbidimetrically at 800/660 nm. None of the patients were currently receiving PCSK9 inhibitor treatment, which would decrease Lp(a) concentration.

### 4.5. Comet Assay

#### 4.5.1. Alkaline Version

The comet assay enables the identification of SSBs and DSBs, as well as alkali labile sites (ALSs). The alkaline version of the comet assay was carried out according to Woźniak et al. [33]. A freshly prepared cell suspension in 0.75% LMP agarose dissolved in PBS was layered onto microscope slides, which was pre-coated with 0.5% NMP agarose. Then, the cells were lysed for 1 h at 4 °C in a buffer containing 2.5 M NaCl, 0.1 M Na2EDTA, 10 mM Tris, 1% Triton X-100, pH 10. After cell lysis, the slides were placed in an electrophoresis unit. DNA was allowed to unwind for 20 min in the solution containing 300 mM of NaOH and 1 mM of Na2EDTA, with a pH > 13.

Electrophoretic separation was performed in the solution containing 30 mM of NaOH and 1 mM of EDTA, with a pH > 13 at an ambient temperature of 4 °C (the temperature of the running buffer did not exceed 12 °C) for 20 min at an electric field strength of 0.73 V/cm (28 mA).

All analyses of DNA damage included a positive control (PC) treated with hydrogen peroxide at 20 μM. The cells were incubated with H_2_O_2_ for 15 min on ice [33].

#### 4.5.2. Comets Assay

After electrophoresis, the slides were washed with deionized water, dried, stained with DAPI at 2 µg/mL, and covered with cover slides. In order to prevent additional DNA damage, this procedure was carried out in limited light or darkness.

One hundred images (comets) were randomly selected from each sample, and the median value of DNA in the comet tail was taken as an index of DNA damage (expressed in percent).

The comets were observed at 200× magnification in an Eclipse fluorescence microscope AXIO SCOPE.A1 (Carl Zeiss, Jena, Germany) attached to an Axiocam 305 color camera (Carl Zeiss, Jena, Germany) equipped with UV-1 filter block (an excitation filter of 359 nm and a barrier filter of 461 nm) (Carl Zeiss, Jena, Germany) and connected to a personal computer-based image analysis system Lucia-Comet v. 7.60 software (Laboratory Imaging, Prague, Czech Republic).

### 4.6. Statistical Analyses

The normality of the distribution of the acquired data was checked using the Shapiro–Wilk test. Some data demonstrated a normal distribution and were analyzed with a parametric test for two dependent groups (paired Student’s *t*-test), while others that were not normally distributed were tested with non-parametric tests for two dependent groups (Wilcoxon test) and for two independent groups (Mann–Whitney test); the choice of test is given in the description of each figure. Statistical analyses were performed with GraphPad Prism 9.0 (GraphPad Software, San Diego, CA, USA). All tests were considered significant at a *p*-value below 0.05.

## 5. Conclusions

iPCSK9 therapy reduces the level of DNA damage in HeFH patients, regardless of the type of inhibitor used. The reduction in DNA damage is most likely associated with the pleiotropic properties of iPCSK9, but the mechanism remains unclear and requires further investigation.

## Figures and Tables

**Table 1:** Characteristics of the subjects with normolipidemia and HeFH.

Parameter Median/(IQR)	Controls, *n* = 20	Patients with HeFH, *n* = 36	*p* Value Control vs. HeFH
Sex, *n* (%)			*p* > 0.05
Female	16 (85%)	22 (61.1%)	
Male	4 (20%)	14 (38.9%)	
Age (y)	48 (33; 53)	57 (45; 68)	*p* < 0.01
Cardiovascular diseases:			
Acute coronary syndrome	0 (0%)	8 (22.2%)	*p* < 0.05
Chronic coronary syndrome	0 (0%)	16 (44.4%)	*p* < 0.001
Lower extremity arterial disease (LEAD)	0 (0%)	5 (13.9%)	*p* > 0.05
Carotid Atherosclerosis (CAS)	0 (0%)	23 (63.9%)	*p* < 0.0001
Stroke	0 (0%)	2 (5.6%)	*p* > 0.05
Myocardial infarction	0 (0%)	5 (13.9%)	*p* > 0.05
Hypertension	0 (0%)	20 (55.6%)	*p* < 0.0001
Diabetes mellitus	0 (0%)	3 (8.3%)	*p* > 0.05
Smoking	0 (0%)	5 (13.9%)	*p* > 0.05
Chronic kidney disease	0 (0%)	4 (11.1%)	*p* > 0.05
Non-alcoholic fatty liver disease	0 (0%)	2 (5.6%)	*p* > 0.05
DCLN score	0 (0%)	22 (61.1%)	N/A
Patients genotype:			
LDLR mutation	0 (0%)	13 (36.1%)	
ApoB mutation	0 (0%)	1 (2.8%)	
PCSK9 mutation	0 (0%)	0 (0%)	
Corneal arcus	0 (0%)	7 (19.4%)	N/A
Tendon Xanthomas	0 (0%)	7 (19.4%)	N/A
Xantelasma	0 (0%)	1 (2.8%)	N/A
Alirocumab	0 (0%)	17 (47.2%)	N/A
Evolokumab	0 (0%)	19 (52.8%)	N/A

**Table 2:** Serum levels of lipid parameters in normolipidemia and HeFH patients before and after treatment with PCSK9 inhibitors.

Lipid Parameters Median (IQR)	Controls, *n* = 20	Patients with HeFH, Before PCSK9i Treatment, *n* = 36	Patients with HeFH, After PCSK9i Treatment, *n* = 36	*p* Value HeFH vs. HeFH on PCSK9i	% Reduction or Increase
Total cholesterol	192.5 (163.8; 229)	235 (179; 275)	130.5 (110.3;170.5)	<0.0001	44.5%
HDL-C	61.7 (48.6; 68.3)	54.3 (44.9; 63)	58.6 (50.2; 67.9)	<0.05	7.3%
Non-HDL	134 (117.5; 156.3)	171.5 (121.5; 220.8)	76 (51; 109)	<0.0001	55.7%
LDL-C	112.5 (95.5; 137)	151.5 (104.3; 196.5)	51.5 (31.7; 70.7)	<0.0001	66%
Triglycerides	131 (84; 194.8)	120.5 (91.5; 172)	94 (75; 132)	<0.01	22%
Lp(a)	10.7 (3.85; 23.9)	120.1 (17; 315.3)	96.7 (8.4; 210.9)	<0.0001	32.9%

## Data Availability

The raw data are shown in Supplementary Material. The sequence data that support the findings of this study have been deposited in the zenodo.org repository with the DOI code 10.5281/zenodo.14190928.

## Supplementary Materials

The following supporting information can be downloaded at: https://www.mdpi.com/article/10.3390/ijms262110529/s1.
